# Family Caregivers of Adults Aged 80 and over: Caregiving as a Stress Process and a Disruption of Occupational Balance

**DOI:** 10.3390/healthcare14101305

**Published:** 2026-05-12

**Authors:** Alice Blin, Sylvie Bonin-Guillaume, Sylvie Arlotto, Stephanie Gentile

**Affiliations:** 1 Service d’Evaluation Médicale, Assistance Publique Hôpitaux de Marseille, 13005 Marseille, France; alice.blin@hotmail.fr (A.B.); sylvie.arlotto@ap-hm.fr (S.A.); 2 Neurosciences of Systems Institute, Institut National de la Santé et de la Recherche Médicale, UMR-Inserm 1106, Aix Marseille University, 13005 Marseille, France; sylvie.bonin@ap-hm.fr; 3 Internal Medicine and Geriatric Department, Hôpitaux Universitaires de Marseille, Assistance Publique Hôpitaux de Marseille, 13005 Marseille, France; 4 School of Medicine, Aix Marseille University, 13005 Marseille, France

**Keywords:** family caregivers, ageing, occupational balance, stress process model, qualitative research, care coordination, case management, older adults

## Abstract

**Background**: Population ageing increases reliance on family caregivers (FCGs) for very old adults (80+). While caregiving is often studied as a source of burden, its impact on caregivers’ daily life and occupational balance remains underexplored. This study aimed to explore how caregiving responsibilities shape the daily lives, occupational balance, and support needs of FCGs, using the Stress Process Model (SPM) and the concept of Occupational Balance (OB). **Methods**: A qualitative study was conducted in the PACA region (France) within the SCOPE project. Seventeen semi-structured interviews were analysed using thematic content analysis, with independent double coding by two researchers. **Results**: Six themes were identified: caregiving role and identity, consequences, occupational patterns, needs, proposed actions, and barriers and facilitators. Caregiving generated both primary stressors (physical and emotional demands) and secondary stressors (role conflicts, financial strain, and social isolation). It also led to occupational imbalance, characterized by reduced leisure, diminished self-care, and reorganization of daily routines. Working FCGs reported greater role strain and time constraints, whereas retired FCGs emphasized informational needs and adaptation strategies. Across both groups, caregivers’ needs were rarely formally assessed. **Conclusions**: These findings highlight that caregiving for very old adults profoundly reshapes caregivers’ daily lives through both stress-related mechanisms and disruptions in occupational balance. They underscore the need for tailored, context-sensitive support strategies, including systematic needs assessment and more structured, individualized coordination approaches such as case management.

## 1. Introduction

Population ageing represents a major public health challenge worldwide [1,2]. In France, projections estimate that by 2060, one in three individuals will be aged 60 or older [3]. This demographic shift implies not only a growing number of older adults but also an increasing reliance on family caregivers (FCGs) to maintain autonomy and quality of life at home [4].

In this article, family caregivers are defined as informal, unpaid relatives or close persons who provide regular emotional, practical, domestic, administrative, financial, or personal support to an older adult experiencing loss of autonomy. In France, several million individuals are involved in such caregiving roles [5]. These responsibilities typically encompass a wide range of tasks, including domestic support, coordination of care, administrative management, emotional support, and assistance with daily living activities.

Throughout this manuscript, the focus is on family caregivers, rather than on the older adults themselves, in order to better understand how caregiving responsibilities affect caregivers’ daily lives and well-being.

While the contribution of FCGs is essential to maintaining older adults’ autonomy, it comes at a cost. Caregiving often entails considerable strain, with consequences for FCGs’ physical and mental health, professional life, and social participation [6,7,8,9]. Previous research has largely focused on caregiver burden, a multidimensional construct describing the stress and exhaustion associated with caregiving [10,11,12]. However, this perspective does not fully capture how caregiving reshapes FCGs’ daily lives and occupations.

In occupational science, occupations refer to meaningful everyday activities that structure daily life and contribute to well-being [13]. When caregiving responsibilities become predominant, they may disrupt this balance, leading to what is termed occupational imbalance [14]. Occupational balance is defined as the subjective perception that one’s daily occupations are harmoniously distributed and satisfying [10]. It reflects not only the time devoted to activities but also their variety, meaning, and the individual’s sense of control [11]. Occupational imbalance has been associated with stress, social isolation, and deteriorating health [10,11,12]. This concept is particularly relevant for FCGs, whose daily routines are often constrained by caregiving demands [14]. It therefore provides a useful framework to understand how caregiving reshapes daily life beyond the notion of burden.

Beyond the notion of burden, caregiving can also be analysed through the Stress Process Model developed by Pearlin et al. [15,16], which conceptualizes caregiving as a dynamic process involving primary and secondary stressors, as well as health-related outcomes. This study adopts a dual conceptual lens: while the Stress Process Model highlights the pathways through which caregiving generates strain, the concept of occupational balance captures the subjective experience of harmony—or imbalance—across daily occupations. Together, these frameworks offer a comprehensive understanding of caregiving as both a stress process and a reorganization of everyday life.

International research shows that FCGs’ needs are heterogeneous and evolve over time, ranging from financial assistance and psychosocial support to improved coordination with professional services [17,18,19,20]. However, these needs are still rarely assessed in a systematic way, leaving many FCGs invisible to health and social care systems. In France, despite recent national strategies to support FCGs [21], evidence remains limited regarding those caring for very old adults (80+), particularly when considering differences between working-age and retired FCGs.

This study focuses on family caregivers of adults aged 80 years and older, rather than on the older adults themselves. It aims to explore how caregiving responsibilities reshape caregivers’ daily occupations and occupational balance, and to identify their needs in relation to their life context. More specifically, the study examines how these experiences differ between working and retired caregivers, in order to better understand the factors shaping caregiving experiences and to inform targeted support strategies.

## 2. Materials and Methods

### 2.1. Study Design

We conducted an exploratory qualitative, non-interventional study in the Provence-Alpes-Côte d’Azur (PACA) region of France between December 2020 and June 2022. This study constitutes the first stage of the SCOPE PACA project, a mixed-methods research program designed to identify the needs of FCGs of older adults and to develop tailored support strategies. The qualitative phase aimed to explore FCGs’ lived experiences in depth, while the subsequent quantitative phase (not reported here) involved a larger survey informed by these findings.

### 2.2. Study Population and Recruitment

Participants were selected from a cohort of family caregivers (FCGs) previously involved in the Carsat study in 2016 [22]. Carsat (Caisse d’Assurance Retraite et de Santé au Travail) is a French social security organization responsible for pension and occupational health. The original cohort included 686 caregivers. Among them, 666 had updated contact details and were considered eligible for recontact. For the present study, Carsat recontacted these individuals by letter and telephone, explaining the aims of the qualitative research.

Inclusion criteria were:

- Being an informal caregiver (family member, spouse, child, or relative) of an older adult aged 80 years or over;
- Providing regular support (emotional, organizational, domestic, financial, or personal care);
- Ability to provide informed consent.

### 2.3. Data Collection

Semi-structured interviews were conducted by AB (Alice Blin), a female researcher trained in qualitative research methods with prior experience in conducting interviews in healthcare research, by telephone or videoconference depending on participants’ preferences and technical availability.

An interview guide, informed by the literature on caregiving and occupational balance [13], was developed by the research team and iteratively refined throughout the data collection process. It covered three main domains: the caregiving role and its implications in daily life (activities, responsibilities, perceptions); perceived impacts on quality of life (physical, psychological, social, and professional); and expressed needs and suggestions for support strategies.

Probing techniques were used to encourage participants to elaborate on concrete experiences. A short socio-demographic questionnaire was also administered, collecting data on age, sex, education level, employment status, relationship to the older adult, and household income.

The research team included professionals from public health and clinical backgrounds, with prior experience in qualitative data collection and analysis. To ensure reflexivity, the researchers engaged in regular discussions throughout the data collection and analysis process to reflect on their assumptions and interpretations.

### 2.4. Ethical Considerations

The study was approved by the Ethics Committee of Aix-Marseille University (No. 2021-02-11-02) and by the Comité de Protection des Personnes (CPP) (No. 21053–39557). Data protection procedures were validated by the French Data Protection Authority (CNIL No. 385-2021). Participants received written information and provided informed verbal consent prior to participation. In accordance with ethical guidelines, written consent was not required.

### 2.5. Data Analysis

All interviews were transcribed verbatim, including relevant non-verbal elements such as pauses and emotional expressions, and anonymized prior to analysis.

Data were analysed using thematic content analysis based on Bardin’s approach [23]. The analysis followed an iterative process involving familiarization with the data, coding of meaningful units, and the development of subthemes and overarching themes through constant comparison. Thematic categorization was guided by the classical principles of content analysis, including homogeneity, exclusivity, and exhaustiveness of categories.

Two researchers (AB and SA), both trained in qualitative methods, independently coded the transcripts and discussed discrepancies until consensus was reached, thereby enhancing analytical rigor. A thematic tree and codebook were developed iteratively to organize categories, subthemes, and illustrative quotes.

Sampling was guided by the principle of theoretical saturation, defined as the point at which no new themes emerged across interviews [24]. Saturation was considered achieved after 17 interviews, as no additional themes or variations were identified in the data.

The reporting of this qualitative study adheres to the Consolidated Criteria for Reporting Qualitative Research (COREQ) guidelines (see Table S1).

Sociodemographic variables were used to describe the sample and to contextualize the qualitative findings. Given the exploratory qualitative design and small sample size, these data are presented descriptively without inferential interpretation.

## 3. Results

**Figure 1:**
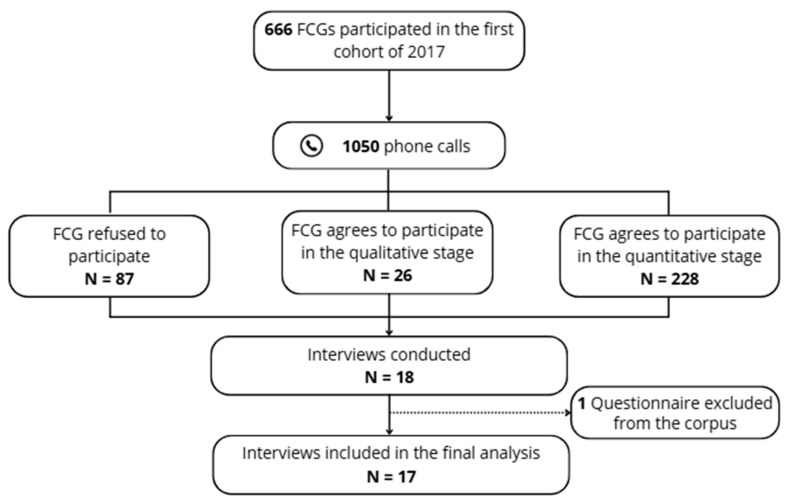
Flow diagram of participant selection and inclusion in the qualitative study.

The diagram summarizes the recruitment process from the initial cohort to the final sample included in the qualitative analysis (Figure 1). Among the 666 individuals with updated contact details, a total of 1050 phone calls were made to contact potential participants, reflecting repeated contact attempts. Of these, 87 declined to participate.

A total of 228 individuals consented to participate in the overall SCOPE project, including 26 who agreed to take part in the qualitative phase. Among these 26 participants, 18 interviews were conducted due to availability constraints, and 17 were included in the final analysis after exclusion of one incomplete interview.

Fifteen interviews were conducted by telephone and two by videoconference. Interview duration ranged from 20 to 90 min (mean: 40 min).

In line with qualitative research standards, data saturation was considered to be reached after 17 interviews, as no new themes or meaningful variations emerged across the topics explored. The research team continuously monitored the emergence of new codes during the analysis, confirming that additional interviews were unlikely to generate further insights.

### 3.1. Participant Characteristics

Seventeen interviews were included in the final analysis. The mean age of FCGs was 63 years (range: 40–87), and the majority were women (n = 13/17). Most participants were adult children (n = 11/17) or spouses (n = 4/17) of the care recipients. Six participants (6/17) cohabited with the older adult, while the others lived separately.

Participants presented heterogeneous profiles in terms of employment status: ten were retired (10/17), six were employed (6/17), and one was unemployed (1/17). Retired FCGs had a mean age of 70 years, whereas working-age FCGs averaged 53 years. The older adults supported had a mean age of 86.7 years (range: 80–99), and two-thirds were women (11/17).

### 3.2. Thematic Findings

Thematic analysis identified six main categories reflecting FCGs’ experiences and needs. Among these, three central dimensions emerged as particularly structuring: the caregiving role and identity, the consequences of caregiving, and occupational patterns, which together reflect the core impact of caregiving on daily life. The other themes—needs, proposed actions, and barriers and facilitators—provide complementary insights into caregivers’ expectations and the conditions shaping their experiences.

#### 3.2.1. Caregiving Role and Identity

A majority of FCGs (12/17) described their role primarily in terms of everyday support tasks—such as shopping, housework, or administrative assistance—rather than as an explicit caregiving identity.

*“I don’t really see myself as a caregiver. I just do what needs to be done for my mother—it’s natural”* (Female, 58, daughter).

A smaller number of participants adopted the caregiver label, particularly when responsibilities became comparable to a second job. This was especially evident among working FCGs, who perceived caregiving as an additional workload competing with professional life.

*“For me it’s like having two jobs. I work all day, and then I come home and continue with everything for my father”* (Male, 49, son).

Retired FCGs, in contrast, tended to minimize the term “caregiver” and framed their role as part of a normal life course, although some acknowledged the intensity of their responsibilities.

*“I wouldn’t say I’m a caregiver, I’m just a wife taking care of her husband. Of course, it takes a lot of energy, but it’s part of our life together”* (Female, 72, spouse).

Working-age FCGs were more likely to describe caregiving as an additional “job,” whereas retired FCGs emphasized continuity with family roles.

This ambivalence in self-identification reflects both cultural norms of family duty and the invisibility of caregiving as a recognized social role. It may also constitute a barrier to accessing support, as individuals who do not identify as caregivers are less likely to seek help or engage with formal services.

#### 3.2.2. Consequences of Caregiving

FCGs reported both positive and negative consequences of caregiving. Some highlighted strengthened relationships with the person they cared for, particularly among working-age FCGs, while others described tensions and deterioration in relationships with certain family members.

*“I think it strengthened my bond with my parents a little more”* (Male, 58, child).

Working-age FCGs frequently reported difficulties related to their professional activity, including repeated adjustments to work schedules, requests for leave, or even withdrawal from employment.

*“How do you expect me to work? I had to give up everything. I’m a consultant by trade. So I worked intermittently, doing one-off assignments, and then little by little I had more time to myself”* (Female, 56, child).

The impact on health and daily life was significant across the sample. Psychological exhaustion was reported by a large proportion of participants (14/17), often associated with anxiety about the future.

*“Well, I do it because it has to be done, but I have to push myself a bit… you know, when you’re not in the best of health… […] Yes, I’m a bit tired, not to say very tired, or even beyond that, because it’s no longer possible”* (Male, 87, husband).

*“I know exactly what to do, but psychologically, I can’t take it anymore”* (Female, 63, child).

*“Sometimes I feel completely drained, like I don’t have energy for myself anymore”* (Male, 64, spouse).

Beyond these impacts, 13 FCGs expressed strong concern about the future of the older adult they were supporting.

*“Right now, that’s what worries me […] when I have to be there in person five days a week, it’s really concerning…”* (Female, 47, spouse).

Overall, these findings highlight the ambivalent nature of caregiving, characterized by a tension between emotional closeness and multidimensional burden affecting professional, social, and health domains.

#### 3.2.3. Occupational Patterns

In terms of occupational patterns, FCGs reported that daily activities were largely dominated by caregiving-related tasks, particularly shopping, housework, and meal preparation. Self-care activities were frequently minimized in favor of domestic and administrative responsibilities, reflecting a clear imbalance in the distribution of occupations.

Leisure activities were more frequently reported by retired FCGs (21 mentions) compared to working FCGs (6 mentions). Moreover, the complete absence of leisure was reported three times more often by working FCGs, highlighting a marked restriction of non-caregiving activities in this group.

This imbalance appeared more pronounced among working FCGs, whose competing professional and caregiving demands led to a sharper reduction in leisure and self-care activities compared to retired participants.

*“I used to go swimming twice a week. Now, between work and caring for my father, I can’t find the time”* (Female, 52, daughter).

Working FCGs described stronger tensions in reconciling professional duties with caregiving, often leading to reduced or abandoned activities. In contrast, retired FCGs emphasized adaptations of daily routines rather than complete withdrawal from activities.

*“I stopped working because she couldn’t tell day from night”* (Female, 47, daughter).

Overall, these findings illustrate a reorganization of daily life characterized by reduced engagement in self-care and leisure activities, consistent with situations of occupational imbalance, particularly among working FCGs.

#### 3.2.4. Identified Needs

FCGs reported a range of unmet needs structured around four main domains. First, material and financial support emerged as a major concern, particularly among working FCGs, reflecting the economic burden associated with caregiving responsibilities.

Second, organizational support was widely emphasized, particularly the need for respite services allowing temporary relief from caregiving duties. Nine participants (9/17) highlighted the need to adapt and secure the living environment of the older adult, indicating practical challenges in daily care management.

Coordination with healthcare professionals constituted a third key domain. FCGs expressed a strong need for improved communication and coordination between care providers. These needs varied according to caregivers’ life context, with working FCGs more frequently emphasizing financial and organizational constraints, while retired FCGs highlighted informational and guidance needs.

*“What we need is not more people, but competent people who coordinate with each other”* (Female, 66, child).

Finally, access to information and tailored advice was frequently reported, especially among retired FCGs, who often felt less familiar with available services and support systems.

*“Actually, what I want is to understand this disease…”* (Female, 47, spouse).

Importantly, most participants reported that their needs had never been formally assessed by a professional, pointing to a lack of recognition and systematic identification of caregivers’ needs within healthcare and social systems.

#### 3.2.5. Identified Measures to Support FCGs

FCGs suggested two main types of measures to support their situation. The first relates to direct support targeting caregivers themselves, including access to psychological counselling and financial recognition of their caregiving role. These measures were perceived as essential to alleviate the emotional and economic burden associated with caregiving.

*“Sometimes I just need someone to talk to, someone who understands what I’m going through”* (Female, spouse).

*“It should be financially recognised, because it takes all our time”* (Female, daughter).

The second type concerns indirect support through improvements in the care of the older adult. Participants emphasized the need for better-trained professionals, simplified administrative procedures, and enhanced coordination between healthcare and social care stakeholders.

*“What we need is for professionals to communicate with each other”* (Female, daughter).

*“When care is better organised and coordinated, it makes a huge difference”* (Female, daughter).

Overall, these proposed measures reflect a dual expectation: to be better supported as individuals while also benefiting from a more efficient and coordinated care system for the older person. These expectations differed slightly across profiles, with working FCGs placing greater emphasis on financial and psychological support, while retired FCGs more often stressed the importance of accessible information and system navigation.

#### 3.2.6. Barriers and Facilitators

All participants identified barriers related to their role as FCGs. The COVID-19 pandemic emerged as a major source of disruption, particularly through service interruptions and increased social isolation.

Information-related barriers were also highlighted, including difficulties in accessing reliable information and limited digital literacy.

Environmental barriers—such as geographic isolation or lack of family support—were reported by 10 participants (10/17).

*“No, I’m all alone… that’s the problem”* (Female, 56, child).

At the same time, FCGs also identified several facilitating factors, including support from family members, proximity to the older adult, and effective communication with healthcare professionals.

*“The doctor who listens and recognizes me as a caregiver—that makes a huge difference”* (Female, 61, daughter).

### 3.3. Comparative Insights: Working vs. Retired FCGS

Key differences and similarities between working and retired FCGs are summarized in Table 1. Overall, working FCGs described caregiving as an additional burden competing with professional responsibilities, leading to significant financial pressures, time constraints, and increased social isolation. In contrast, retired FCGs more often framed caregiving as a continuation of their life course, with a greater capacity to adapt their routines and maintain social connections.

These differences were particularly evident in the nature of expressed needs and the actions envisaged to address them. Working FCGs predominantly emphasized financial and organizational support, whereas retired FCGs highlighted the need for information, guidance, and understanding of the care situation.

In contrast, perceived barriers and facilitators appeared largely similar across both groups, suggesting that, despite differing life contexts, FCGs share common structural and relational challenges in their caregiving experience.

Overall, these findings point to two distinct underlying dynamics: caregiving as role overload among working FCGs, and caregiving as role adaptation among retired FCGs.

## 4. Discussion

This qualitative study explored the experiences and needs of family caregivers of very old adults (80+) in the PACA region of France. By framing the findings within both the Stress Process Model (SPM) [15] and the concept of Occupational Balance (OB) [13,14,25,26], our study provides a comprehensive understanding of how caregiving simultaneously generates stressors and disrupts the balance of daily occupations.

### 4.1. Caregiving as a Stress Process

According to Pearlin’s SPM, caregiving involves primary stressors (direct care tasks and physical demands) and secondary stressors (role conflicts, professional constraints, and social isolation), which together impact caregivers’ health and well-being [15].

Our findings clearly reflect this process. FCGs reported exhaustion, anxiety, and a sense of burden as primary stressors, alongside secondary stressors such as difficulties balancing work and caregiving, financial strain, and reduced leisure time. These results are consistent with previous studies demonstrating that the accumulation of stressors increases caregivers’ vulnerability [6,8,19].

Working FCGs, in particular, experienced pronounced tensions between employment and caregiving responsibilities, illustrating the “role strain” dimension of the SPM. In contrast, retired FCGs, although less exposed to work-related conflicts, faced secondary stressors related to limited access to information and lower health literacy, which hindered their ability to navigate available support systems. Across participants, psychological exhaustion and role conflict emerged as the most salient stressors, suggesting that these dimensions play a central role in shaping caregivers’ overall experience.

### 4.2. Occupational Balance and the Reorganization of Daily Life

While the SPM highlights stress mechanisms, the concept of OB offers a complementary perspective by capturing how caregiving reshapes daily life beyond stress alone. OB refers to the subjective experience of having a balanced and meaningful distribution of daily activities, including self-care, productivity, and leisure [13,14,25,26].

Our findings point to clear situations of occupational imbalance, characterized by reduced or abandoned leisure activities, diminished self-care, and an overload of caregiving-related tasks. Working FCGs frequently described a sense of time scarcity, often sacrificing social and leisure activities, whereas retired FCGs emphasized the progressive adaptation of their routines.

These findings are in line with occupational science research showing that imbalance—whether due to overload, monotony, or loss of meaningful activities—is associated with poorer mental health outcomes [26,27].

By introducing the concept of occupational balance into the analysis of caregiving, this study shifts the perspective from burden alone toward a more holistic understanding of caregiver well-being. It suggests that effective interventions should not only reduce stress but also support caregivers in maintaining or restoring meaningful daily activities, particularly among working caregivers who appear most exposed to occupational imbalance.

### 4.3. Contributions and Originality

This study contributes to the literature in three main ways.

First, it focuses specifically on caregivers of very old adults (80+), a population that remains understudied despite facing distinct challenges, including higher levels of frailty among care recipients and the advanced age of caregivers themselves.

Second, it combines two complementary conceptual frameworks—SPM and OB—highlighting caregiving as both a stress-generating process and a disruption of occupational balance.

Third, it provides a comparative perspective between working and retired FCGs, revealing distinct patterns of needs and adaptation. These findings suggest that caregiver support should be tailored according to life stage and employment status. Taken together, these contributions highlight the importance of considering caregiving as both a structural constraint and a lived experience shaped by caregivers’ life context.

### 4.4. Strengths and Limitations

This study has several strengths. First, it focuses on an understudied population—family caregivers of very old adults (80+)—providing insights into a group facing specific and often overlooked challenges. Second, the use of a dual conceptual framework combining the Stress Process Model and Occupational Balance offers a more comprehensive understanding of caregiving, bridging stress-related mechanisms and daily life organization. Third, the qualitative design, supported by in-depth interviews and thematic analysis with double coding, enhances the richness and credibility of the findings.

However, some limitations should be acknowledged. The sample size, although sufficient to reach thematic saturation, remains limited and may not capture the full diversity of caregiving situations. Participants were recruited within the SCOPE project, which may introduce a selection bias toward individuals already engaged or more aware of caregiving issues. In addition, the overrepresentation of women reflects the reality of caregiving but may limit the exploration of gender-specific experiences. Finally, the cross-sectional design does not allow for capturing the dynamic evolution of caregiving over time. Moreover, as data were based on self-reported interviews, the findings may be subject to social desirability bias, with participants potentially under- or over-reporting certain aspects of their caregiving experience.

### 4.5. Implications for Policy and Practice

At the system level, strengthening coordination between health and social care actors appears essential, as fragmentation of services was identified as a major barrier by participants. However, our findings suggest that coordination alone may be insufficient when it remains diffuse and poorly operationalized.

FCGs described complex and fragmented care pathways, with multiple actors and frequent breakdowns in communication, often placing the burden of coordination on caregivers themselves. In this context, the identification of a single point of contact—such as a case or care manager—appears crucial to support caregivers. Such a professional could guide FCGs through available services, ensure continuity of care, and facilitate navigation across systems, thereby reducing the organizational burden placed on caregivers.

These findings are consistent with previous studies highlighting the burden of care coordination on informal caregivers and the need for clearer system navigation [18,19,20]. Taken together, they suggest that beyond general coordination efforts, more individualized and clearly assigned case-management approaches may represent a more effective and pragmatic response to system fragmentation.

## 5. Conclusions

This study highlights that caregiving for very old adults profoundly reshapes caregivers’ daily lives through both stress-related mechanisms and disruptions in occupational balance.

By combining the Stress Process Model and the concept of Occupational Balance, the findings show that caregiving is not only a source of primary and secondary stressors, but also a major reorganization of daily life that may lead to occupational imbalance.

Working FCGs were particularly exposed to role conflicts, financial strain, and loss of leisure, whereas retired FCGs emphasized difficulties in accessing information and adapting their routines. Across both groups, caregivers’ needs were rarely formally assessed, reflecting their persistent invisibility within health and social care systems.

These findings suggest that caregiver support should extend beyond burden reduction to actively restoring occupational balance, enabling caregivers to maintain meaningful and diverse daily activities alongside their caregiving role. This implies the need for systematic needs assessments, tailored support strategies according to life stage, and greater recognition of occupational balance as a key determinant of health.

Ultimately, supporting family caregivers is not only essential for their own well-being, but also for preserving the autonomy and quality of life of the very old adults they support.

## Figures and Tables

**Table 1:** Contrasting Caregiving Dynamics in Working and Retired Family Caregivers: Needs, Challenges, and Adaptive Strategies.

Dimension	Working FCGs	Retired FCGs
Caregiving experience	Perceived as an additional “job”; tension with professional responsibilities	Integrated into life course; framed as continuity of family role
Main challenges	Financial pressure; time constraints; work–care conflict (leave, reduced activity)	Managing routines; adapting to caregiving demands
Social impact	Higher levels of social isolation; reduced leisure activities	Better maintenance of social ties; more frequent leisure activities
Health impact	Psychological exhaustion; stress linked to role overload	Fatigue present but often normalized within daily life
Needs expressed	Financial support; organizational support (respite, coordination)	Information and guidance; understanding disease and care pathways
Proportion reporting informational needs	1/3 of participants	All participants
Actions envisaged	Practical and structural support (financial aid, respite services)	Informational and adaptive strategies
Barriers	Time constraints; work-related constraints; organizational complexity	Similar barriers (information access, isolation), but less linked to employment
Facilitators	Support from professionals and relatives when available	Same facilitators (family support, proximity, communication with professionals)

## Data Availability

Due to the qualitative nature of this study and ethical restrictions related to participant confidentiality, raw data (interview transcripts) cannot be publicly shared. Data are available from the corresponding author upon reasonable request.

## Supplementary Materials

The following supporting information can be downloaded at: https://www.mdpi.com/article/10.3390/healthcare14101305/s1, Table S1: COREQ (COnsolidated criteria for REporting Qualitative research) Checklist. Ref. [28] was cited in the Supplementary Materials.
